# Supplementary material for: HIVprotI: an integrated web based platform for prediction and design of HIV proteins inhibitors
Source: J Cheminform. 2018 Mar 9;10:12. doi: 10.1186/s13321-018-0266-y (PMC5845081; doi:10.1186/s13321-018-0266-y)
Supplement: Supplementary file 2 — Additional file 2. Source code of HIVProtI web server. [file 13321_2018_266_MOESM2_ESM.zip › HIVprotI_Source-code/hivprotI/weka-3-6-6/doc/weka/gui/beans/BatchClustererListener.html]

BatchClustererListener


---


|  |  |  |
| --- | --- | --- |
| **Overview** \| **Package** \| **Class** \| **Tree** \| **Deprecated** \| **Index** \| **Help** \| **Weka's home** | |  |
| **PREV CLASS**   **NEXT CLASS** | **FRAMES**    **NO FRAMES**     **All Classes** |  |
| SUMMARY: NESTED \| FIELD \| CONSTR \| METHOD | DETAIL: FIELD \| CONSTR \| METHOD |  |


---


## weka.gui.beans Interface BatchClustererListener

**All Superinterfaces:**: java.util.EventListener

**All Known Implementing Classes:**: ClustererPerformanceEvaluator, PredictionAppender, SerializedModelSaver

---

``` public interface BatchClustererListener extends java.util.EventListener ```

Interface to something that can process a BatchClustererEvent

**Version:**
:   $Revision: 1.2 $

**Author:**
:   MStefan Mutter

**See Also:**: `EventListener`

---

| **Method Summary** | |
| --- | --- |
| `void` | `acceptClusterer(BatchClustererEvent e)`             Accept a BatchClustererEvent |

**Method Detail**

### acceptClusterer

```
void acceptClusterer(BatchClustererEvent e)
```

:   Accept a BatchClustererEvent

    :   **Parameters:**: `e` - a `BatchClustererEvent` value


---


|  |  |  |
| --- | --- | --- |
| **Overview** \| **Package** \| **Class** \| **Tree** \| **Deprecated** \| **Index** \| **Help** \| **Weka's home** | |  |
| **PREV CLASS**   **NEXT CLASS** | **FRAMES**    **NO FRAMES**     **All Classes** |  |
| SUMMARY: NESTED \| FIELD \| CONSTR \| METHOD | DETAIL: FIELD \| CONSTR \| METHOD |  |


---
